# VenomMaps: Updated species distribution maps and models for New World pitvipers (Viperidae: Crotalinae)

**DOI:** 10.1038/s41597-022-01323-4

**Published:** 2022-05-25

**Authors:** Rhett M. Rautsaw, Gustavo Jiménez-Velázquez, Erich P. Hofmann, Laura R. V. Alencar, Christoph I. Grünwald, Marcio Martins, Paola Carrasco, Tiffany M. Doan, Christopher L. Parkinson

**Affiliations:** 1 Clemson University, Department of Biological Sciences, Clemson, SC USA; 2 Universidad Nacional Autónoma de México, Posgrado en Ciencias Biológicas, Mexico City, Mexico City Mexico; 3 Vida Silvestre Coatl AC, Mexico City, Mexico City Mexico; 4 Cape Fear Community College, Science Department, Wilmington, NC USA; 5 HERP.MX, Villa del Álvarez, Colima Mexico; 6 Universidade de São Paulo, Departamento de Ecologia, Instituto de Biociências, São Paulo, São Paulo Brazil; 7 Universidad Nacional de Córdoba, Facultad de Ciencias Exactas, Físicas y Naturales, Centro de Zoología Aplicada, Córdoba, Argentina; 8 Consejo Nacional de Investigaciones Científicas y Técnicas (CONICET), Instituto de Diversidad y Ecología Animal (IDEA), Córdoba, Argentina; 9 New College of Florida, Division of Natural Sciences, Sarasota, FL USA; 10 Clemson University, Department of Forestry and Environmental Conservation, Clemson, SC USA

**Keywords:** Biogeography, Biodiversity

## Abstract

Beyond providing critical information to biologists, species distributions are useful for naturalists, curious citizens, and applied disciplines including conservation planning and medical intervention. Venomous snakes are one group that highlight the importance of having accurate information given their cosmopolitan distribution and medical significance. Envenomation by snakebite is considered a neglected tropical disease by the World Health Organization and venomous snake distributions are used to assess vulnerability to snakebite based on species occurrence and antivenom/healthcare accessibility. However, recent studies highlighted the need for updated fine-scale distributions of venomous snakes. Pitvipers (Viperidae: Crotalinae) are responsible for >98% of snakebites in the New World. Therefore, to begin to address the need for updated fine-scale distributions, we created VenomMaps, a database and web application containing updated distribution maps and species distribution models for all species of New World pitvipers. With these distributions, biologists can better understand the biogeography and conservation status of this group, researchers can better assess vulnerability to snakebite, and medical professionals can easily discern species found in their area.

## Abstract (summary)

**Table:** 

Measurement(s)	Species Distributions
Technology Type(s)	Geographic Information System • Species Distribution Model (MaxEnt/kuenm)
Factor Type(s)	Occurrence Records • Environmental Data
Sample Characteristic - Organism	Crotalinae
Sample Characteristic - Location	North America • South America

## Background & Summary

Knowing where a species occurs is critical for understanding numerous aspects of biology including evolution, biogeography, ecology, and conservation. Species distributions are also important to applied disciplines outside biology. The general public and citizen scientists utilize published distributions in field guides to assist with identification, government agencies utilize known distributions to better plan management strategies, and medical practitioners must maintain a working knowledge of dangerous taxa in their area to better treat patients afflicted during an encounter. One group which highlights the importance of having well-described distributions is venomous snakes. Snake venom can vary tremendously both between and within a single species^1–4^ and envenomation by venomous snakes (hereafter, snakebite) is regarded as a priority neglected tropical disease by the World Health Organization due to the nearly 100,000 deaths and 400,000 disablements that occur globally every year^5^. Therefore, knowing which venomous snake species occur in a given area can inform snakebite risk analyses and inform medical treatment of snakebite^6–8^.

Venomous snakes have a cosmopolitan distribution, occurring on every continent but Antarctica. Most medically significant species (*i.e*., those resulting in hospitalization, permanent injury, or death to humans) fall into one of two families: Viperidae (vipers) and Elapidae (elapids). Other families such as Atractaspididae, Colubridae, and Dipsadidae also contain species capable of inflicting medically significant bites; however, they make up a small proportion of envenomations compared to vipers and elapids^9–12^. In general, vipers contribute the most snakebites globally^9–12^, particularly in North and South America (*i.e*., the New World) where pitvipers (subfamily Crotalinae) such as rattlesnakes (*Crotalus* and *Sistrurus*), cantils (*Agkistrodon*), and lanceheads (*Bothrops*) are responsible for more than 98% of envenomations^9,11,12^. Many species of New World pitvipers exhibit functional venom variation both between species and within species across geographic space^1–4^ which impacts snakebite treatment. As such, clear delimitation of species’ ranges can inform medical treatment and antivenom use to better account for interspecific venom variation.

Unfortunately, the distributions of many species of venomous snakes remain unrefined, impacting our biological understanding of these organisms and limiting snakebite epidemiology. The need for refined species distributions of venomous species is well-documented, even in thorough studies synthesizing species distributions with epidemiological data. Hansson *et al*.^7^ used snake distributions to identify areas in need of improved accessibility to antivenom in Costa Rica^7^. Similarly, Yañez-Arenas *et al*.^6^ demonstrated that species distribution models were able to explain up to 35% of the variation in the incidence of snakebites across Veracruz, Mexico and could be used to infer potential areas of high snakebite risk. A more recent study used venomous snake distributions and occurrence records to map global vulnerability to snakebite envenoming based on antivenom availability, hospital accessibility, and the Healthcare Access and Quality (HAQ) index^8^. Each of these studies was limited by the available digitized distribution information^6–8^. For example, Longbottom *et al*.^8^ excluded nine species due to an absence of geographical information, identified 216 species that require distributional assessments, and emphasized the importance of and need for fine-scale (≤10 km^2^) distributional information for venomous snake species^8^.

Here, we focus on enhancing fine-scale distributional information for venomous snakes by compiling novel distribution maps and species distribution models (SDMs) for all 158 species of pitvipers in North, Central, and South America in an easy to use, publicly available web interface. We use occurrence records, published distribution maps, ecoregion maps, and a relief map to manually reconstruct the distributions of these species. Species Distribution Modeling (SDM) and Ecological Niche Modeling (ENM) are also often used to estimate the geographic ranges. Although there are theoretical differences between these two tools, both utilize niche theory to model processes that shape distributions based on statistical associations between environmental predictors and records of species presence^13,14^. Therefore, in addition to our curated distribution maps, we perform species distribution modeling to capture fine-scale (1 km^2^) distribution information that may be missed in our hand-curated, generalized distributions. We chose SDM because our goal is to map the species distribution rather than understand the processes/factors underlying the niche. This dataset includes approximately 74 of the 216 species of venomous snakes identified by Longbottom *et al*.^8^ as needing reassessment and adds 69 additional species that were either recently described or did not have prior distribution information and were thus excluded in these analyses^8^. These distribution maps can be used for a variety of purposes including snakebite vulnerability assessment, informing medical treatment via species identification, assessing access to appropriate antivenom, biogeographic analyses, conservation assessments, and for general information on species ranges. Finally, we provide all the code and methodologies used for range estimation and the final maps in an intuitive user-friendly, publicly-accessible GitHub repository (github.com/RhettRautsaw/VenomMaps) and Shiny application (rhettrautsaw.app/shiny/VenomMaps) available from any computer with an internet connection. Stable releases are archived on Zenodo^15^.

## Methods

The custom code used to clean occurrence records and construct SDMs is available at (github.com/RhettRautsaw/ VenomMaps). We used the following R^16^ packages for data cleaning, manipulation, species distribution modeling, and Shiny app creation: *tidyverse*^17^ *readxl*^18^, *data.table*^19^, *sf*^20^, *sp*^21,22^, *rgdal*^23^, *raster*^24^, *smoothr*^25^, *ape*^26^, *phytools*^27^, *argparse*^28^, *parallel*^16^, *memuse*^29^, *dismo*^30^, *rJava*^31^, *concaveman*^32^, *spThin*^33^, *usdm*^34^, *ENMeval*^35^, *kuenm*^36^, *shiny*^37^, *leaflet*^38^, *leaflet.extras*^39^, *leaflet.extras2*^40^, *RColorBrewer*^41^, *ggpubr*^42^, *ggtext*^43^, and *patchwork*^44^.

### Updating occurrence record taxonomy

Our goal was to update and reconstruct the distributions of New World pitvipers. We used the Reptile Database^45^ (May 2021) as our primary source for current taxonomy which included the following genera: *Agkistrodon, Atropoides, Bothriechis, Bothrocophias, Bothrops, Cerrophidion, Crotalus, Lachesis, Metlapilcoatlus, Mixcoatlus, Ophryacus, Porthidium*, and *Sistrurus*. However, to ensure we captured all New World pitvipers records, we incorporated all members of the family Viperidae (all vipers and pitvipers) into our pipeline for updating occurrence record taxonomy (*i.e*., to account for errors in the recorded latitude, longitude, or if subfamily was not recorded).

First, we collected global occurrence records for “Viperidae” from GBIF (downloaded 2021-08-19)^46^, Bison (downloaded 2021-08-19)^47^, HerpMapper (only New World taxa; downloaded 2021-08-19)^48^, Brazilian Snake Atlas^49^, BioWeb (downloaded 2021-07-07)^50^, unpublished data/databases from RMR, GJV, EPH, LRVA, MM, and CLP, and georeferenced literature records totaling 373,673 species-level records, 292,425 of which are New World pitvipers. Given the fluidity of taxonomy, records were often associated with outdated names. For example, *Crotalus mitchelli pyrrhus* was elevated to *Crotalus pyrrhus*^51^, but may still be recorded as the former in a given repository (*e.g*., GBIF). To correct taxonomy in our database, we checked records against a list of synonyms found on the Reptile Database and compared them to current taxonomy. If species and subspecies columns matched the same taxon (or no subspecies was recorded), then species IDs were not altered. If species and subspecies IDs did not match the same taxon, we updated taxonomy by minimizing the number of changes required to a given character string. We then manually checked all changes.

### Constructing distribution maps

Next, we collected preliminary distribution maps from the International Union for Conservation of Nature (IUCN; downloaded 2018-11-27)^52^, Global Assessment of Reptile Distributions (GARD) v1.1^53^, Heimes^54^, Campbell and Lamar^55^, and unpublished maps. We manually curated distribution maps for all New World pitvipers in QGIS using the occurrence records, previous distribution maps, and recent publications for each taxon (note that distributions for Old World Viperidae have not yet been updated). We used a digital relief map (maps-for-free.com) and The Nature Conservancy Terrestrial Ecoregions (TNG.org)^56^ to identify clear distribution boundaries (*e.g*., mountains). We then clipped the final distributions to a land boundary (GADM v3.6)^57^ and smoothed the distribution using the the “chaikin” method in the R package *smoothr*^25^.

### Occurrence-distribution overlap

Our initial taxonomy check was only concerned with records for which a subspecies was recorded and had since been elevated to species status. Therefore, many records with no assigned subspecies likely remained associated with an incorrect or outdated generic and/or specific identification. Fortunately, taxonomic changes are typically associated with changes in the species’ expected distribution. For example, when *Crotalus simus* was resurrected from *C. durissus*, the distribution of *C. durissus* was split: the northern portion of its range in Central America now represented the resurrected species (*C. simus*) and the southern portion of its range remained *C. durissus*^55^. Yet, occurrence records in Central America often remain labelled as *C. durissus* in data repositories. Therefore, we spatially joined records with the newly reconstructed species distribution maps to determine if they overlapped with their expected distribution (Old World taxa were joined with the GARD 1.1 distributions^53^).

Briefly, we developed a custom function (occ_cleaner.R) to perform the spatial join and update taxonomy. First, we calculated the distance for each record to the 20 nearest distributions within 50 km (full overlap resulted in a distance of 0 m). Next, we calculated the phylogenetic distance between the recorded species ID and each species with which that record overlapped using the tree from Zaher *et al*.^58^ and adding taxa based on recent clade-specific publications (bind.tip2.R; see github.com/RhettRautsaw/VenomMaps for full list of references and details). If records overlapped with their expected species, no changes were made. If records fell outside of their expected distribution, we filtered the potential overlapping and nearby species (within 50 km) to minimize phylogenetic distance. If multiple species were equally distant (*i.e*., share the same common ancestor), we attempted to minimize geographic distance. If multiple species remained equally distant in both phylogenetic and geographic distance, we flagged the record to be manually checked. We also flagged records if a species’ taxonomy had changed and records were additionally flagged as potentially dubious if the taxonomic change had a phylogenetic divergence greater than 5 million years. We manually checked all flagged records and returned records to their original species ID if species identity remained uncertain. We flagged these records as potentially dubious, along with records that fell outside of their expected distribution (within 50 km), and removed all flagged records for species distribution modeling. Our final cleaned database contained 344,998 global records, of which 275,087 were New World pitvipers.

### Species distribution modeling

We attempted to infer SDMs for the 158 species of New World pitvipers currently recognized by the Reptile Database (May 2021) and additionally modeled the three subspecies of *Crotalus ravus* separately based on recommendations for species status elevation by Blair *et al*.^59^ for a total of 160 species. We developed a unix-executable R script (autokuenm.R) designed to take occurrence records, distribution maps, and environmental data and prepare these data for species distribution modeling with *kuenm*^36^. We chose to use *kuenm* – and MaxEnt v3.4.4^60^ – because it has been shown to have good predictive power^61^ and fine-tuning of this algorithm has performance comparable to more computationally intensive ensembles^62,63^. Additionally, MaxEnt allows for flexibility in parameter selection^64^ and can function entirely with presence data^14^.

Prior to autokuenm, to account for sampling/spatial bias during SDM, we created a bias file by using the pooled New World pitviper occurrence records as representative background data^65–68^. Specifically, we converted occurrence records to a raster and performed two-dimensional kernel density estimation (kde2d) with the *MASS* package with default settings^69^ and rescaled the kernel density by a factor of 1000 and rounded to three decimal places. This was then used as input to factor out sampling bias by MaxEnt. We then ran autokuenm, which is designed to subset/partition the cleaned occurrence records for a given species and prepare additional files for SDM. We first defined M-areas – or areas accessible to a given species – using the World Wildlife Fund Terrestrial Ecoregions^70^. Biogeographic regions represent distributional limits for many species and are reasonable hypotheses for the areas accessible to a given species^71,72^. To do this, we created alpha hulls from the subset of occurrence records for a given species using concaveman^32^ with default settings. We then identified regions with at least 20% of the region covered by the alpha hull and merged these regions together to form our final M-area. All environmental layers and the bias file were cropped to this M-area which was used as the geographic extent for modeling. We then randomly selected 5% of records to function as an independent test set for final model evaluation. Next, we generated 2000 random background points across the cropped environmental layers and used ENMeval to partition occurrence records into four sets using the checkerboard2 pattern^35^. Note that the background points here were not used in MaxEnt. One of the four partitions was selected at random to be used as the testing set; the remaining three partitions were used for training the MaxEnt models. If the number of occurrence records in the independent test set was less than five, then we used the training partition for final model creation and used the testing partition for final model evaluation.

We tested the top-contributing variables from three sets of environmental layers: (1) bioclimatic variables, (2) EarthEnv topographic variables^73^, and (3) a combination of these variables. To select the top-contributing variables in each set, we wrote a custom function (SelectVariables) which used a combination of MaxEnt permutation importance and Variable Inflation Factors (VIF) to remove collinearity while keeping the variables that contributed the most to the model. Compared with variable selection via principal component analysis loadings, the permutation importance and VIF methodology demonstrated significant improvement in MaxEnt model fit. First, we designed SelectVariables to run MaxEnt using *dismo::maxent* with default settings and then extracted the permutation importance. We removed variables if they had 0% permutation importance. Next, we calculated VIF with *usdm::vif* and then iteratively removed variables by selecting the variables with two highest VIF values and removing whichever variable had the lowest permutation importance. We then recalculated VIF and repeated the process until the maximum VIF value was less than 10. Finally, we recalculated permutation importance with the remaining variables using *dismo::maxent* with default settings and removed variables with less than 1% permutation importance to create the final variable sets. This process was done for each species independently.

With the final environmental variable, testing, and training sets, we generated SDMs using *kuenm*. First, we created candidate calibration models with multiple combinations of regularization multipliers (0.1, 0.2, 0.3, 0.4, 0.5, 0.6, 0.7, 0.8, 0.9, 1, 2, 3, 4, 5, 6, 8, 10), feature classes (l, q, h, lq, lp, lt, lh, qp, qt, qh, pt, ph, th, lqp, lqt, lqh, lpt, lph, lth, qpt, qph, qth, pth, lqpt, lqph, lqth, lpth, qpth, lqpth), and sets of environmental predictors (bioclimatic, topographic, combination) totaling 2,958 candidate models per species. We then ran each model in parallel using GNU Parallel^74^. Next, we evaluated the candidate models and selected the best models using statistical significance (partial ROC), prediction ability (omission rates; OR), and model complexity (AICc) with the “*kuenm_ceval*” function with default settings. Specifically, models were only considered if they were statistically significant and had an OR less than 5%. If no models passed the OR criteria, the models with the minimal OR were considered. Finally, any remaining models were filtered to those within 2 AICc of the top model (Supplementary Table 1). In addition to evaluating and comparing all models together, we evaluated bioclimatic-only and combination-only models separately since these two sets of environmental variables were expected to be the best performing models given the ubiquity of bioclimatic variables in species distribution modeling (Supplementary Table 1).

We generated 10 bootstrap replicates for each of the “best” calibration models using the “*kuenm_mod*” function. We also performed jackknifing to assess variable importance and models were output in raw format. We evaluated the final models using “*kuenm_feval*” with default settings. To select the best model for each comparative set (*i.e*., all, bioclimatic-only, and combination-only sets), we filtered the final evaluation results to minimize the OR and maximize the AUC ratio (Supplementary Table 2). If multiple models remained and were considered equally competitive, we averaged these models together (Supplementary Table 3). Because we performed three different set of comparisons, there were three “best” models per species, so we again aimed to minimize the OR and maximize the AUC ratio to select a final model for each species (Supplementary Table 4). We then converted our final models into cloglog format for visualization and threshold the models using a 10th percentile training presence cutoff (Fig. S2). Both conversion and thresholding functions are provided as R functions (raw2log, raw2clog, raster_threshold in functions.R; github.com/RhettRautsaw/VenomMaps).

## Data Records

A project repository containing all data is available on GitHub (github.com/RhettRautsaw/VenomMaps) with stable releases archived in Zenodo^15^. All distribution maps are available as vector-based geojson files in the project repository under the data/distributions directory. All cleaned occurrence records are available as Microsoft Excel files in the project repository under the data/occurrence directory. All final SDMs are available as raster .tif files under the data/enm directory. This work is licensed under a CC BY 4.0 license.

### Updates

Distributions for Old World Viperidae are currently provided via GARD 1.1^53^; however, the methodology and code used here (*i.e*., VenomMaps) can be made the standard for range estimation and understanding species distributions. We hope to expand this dataset in the future to update the distributions of Old World Viperidae and incorporate other medically significant snake families. We plan to push updates annually and welcome contributions from other experts in the field.

## Technical Validation

### SDM evaluation

**Fig. 1:**
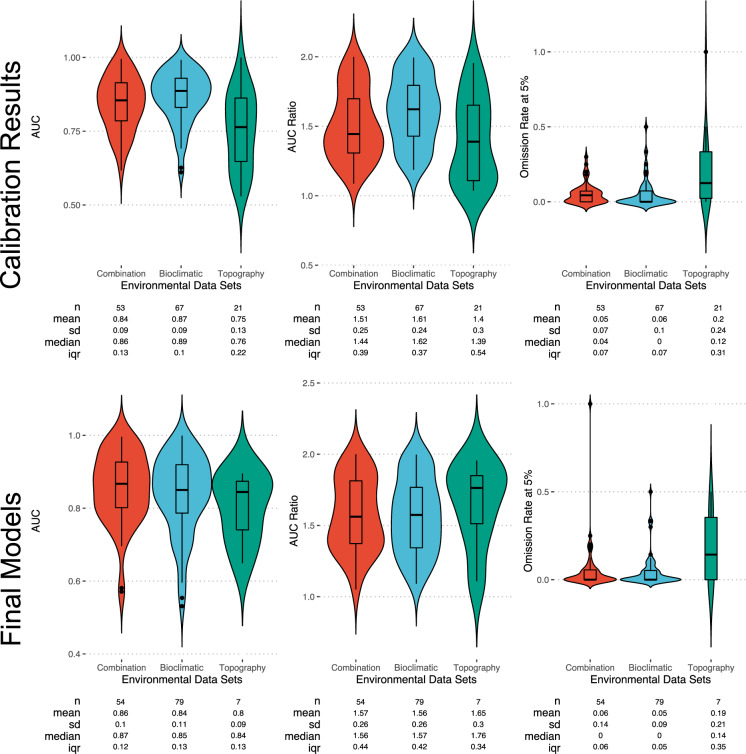
Area Under the Curve (AUC), AUC ratios, and Omission Rates for SDMs. Top row represents the best models selected after *kuenm* calibration of 2,958 models. Bottom row represents the selected final or averaged models for each species.

Of the 160 species of New World pitvipers (including recommendations from Blair *et al*. 2018), 20 had insufficient occurrence records (1–26 records) or occupied a limited geographic area (*e.g*. insular taxa) and species distribution modeling could not be performed (Supplementary Table 4). When comparing all possible model sets, *kuenm* selected a combination of variables as the best model set for 53 species, bioclimatic-only for 67 species, and topography-only for 21 species (Supplementary Table 2). Model Area Under the Curve (AUC) ratios ranged from 1.03–1.99 with a median of 1.53 (Fig. 1; Supplementary Table 2). Topography-only models generally had lower AUC ratios (median = 1.39), compared to bioclimatic-only and combination models (median = 1.62 & 1.44, respectively; Supplementary Table 2). Additionally, when topography was selected, models generally had a high suitability across the entire modeling extent (Fig. S1; Supplementary Table 2). Omission rates were similarly lower for bioclimatic and combination models (median = 0.00 & 0.04, respectively) compared to topographic models (median = 0.12). Topography-only models were generally only chosen when species had a low number of occurrence records (${\bar{x}}_{{\rm{topographic}}}$ = 295; ${\bar{x}}_{{\rm{bioclimatic}}}$ = 386; ${\bar{x}}_{{\rm{combination}}}$ = 892; Supplementary Table 2). Given the poorer fit of topography-only models despite being selected in the comparison of all models, we also performed comparisons of bioclimatic-only and combination-only models separately.

After comparing bioclimatic-only and combination-only models, the selected models in these comparisons often had lower omission rates and higher AUC ratios than the comparison of all models. Therefore, we selected the model from across the three comparative sets which minimized omission rate and maximized AUC Ratio as our final model. In the final model set, a combination of bioclimatic and topographic variables was selected as the best model for 54 species, bioclimatic-only variables was selected as the best model for 79 species, and 7 species remained supporting topographic-only models (Supplementary Table 3; Supplementary Table 4). AUC ratio ranged from 1.04–1.99 with a median of 1.58 and omission rates ranged from 0.00 to 0.14 with a median of 0.00 (Fig. 1; Supplementary Table 3; Supplementary Table 4).

**Fig. 2:**
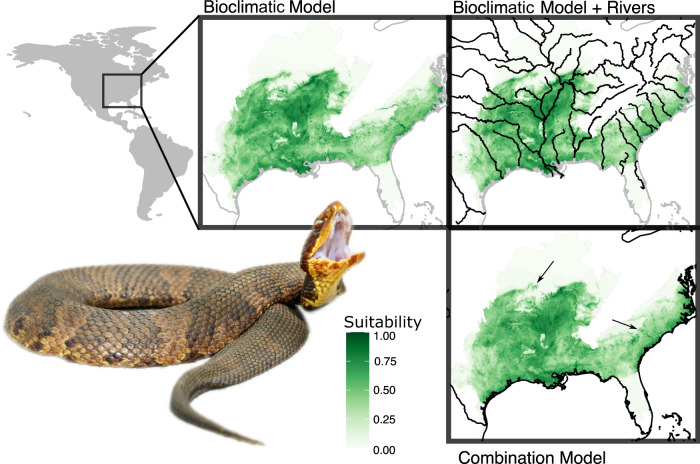
Bioclimatic and Combination SDMs for *Agkistrodon piscivorus* demonstrate how inclusion of additional variables (other than bioclimatic) can refine SDMs to more produce a more precise map of expected habitats for a given species, such as rivers. Arrows in the combination model point to areas where the combination model refines the bioclimatic model. Photo Credit: *A. piscivorus* (No copyright; Jesus Moreno, United States Fish and Wildlife Service).

**Fig. 3:**
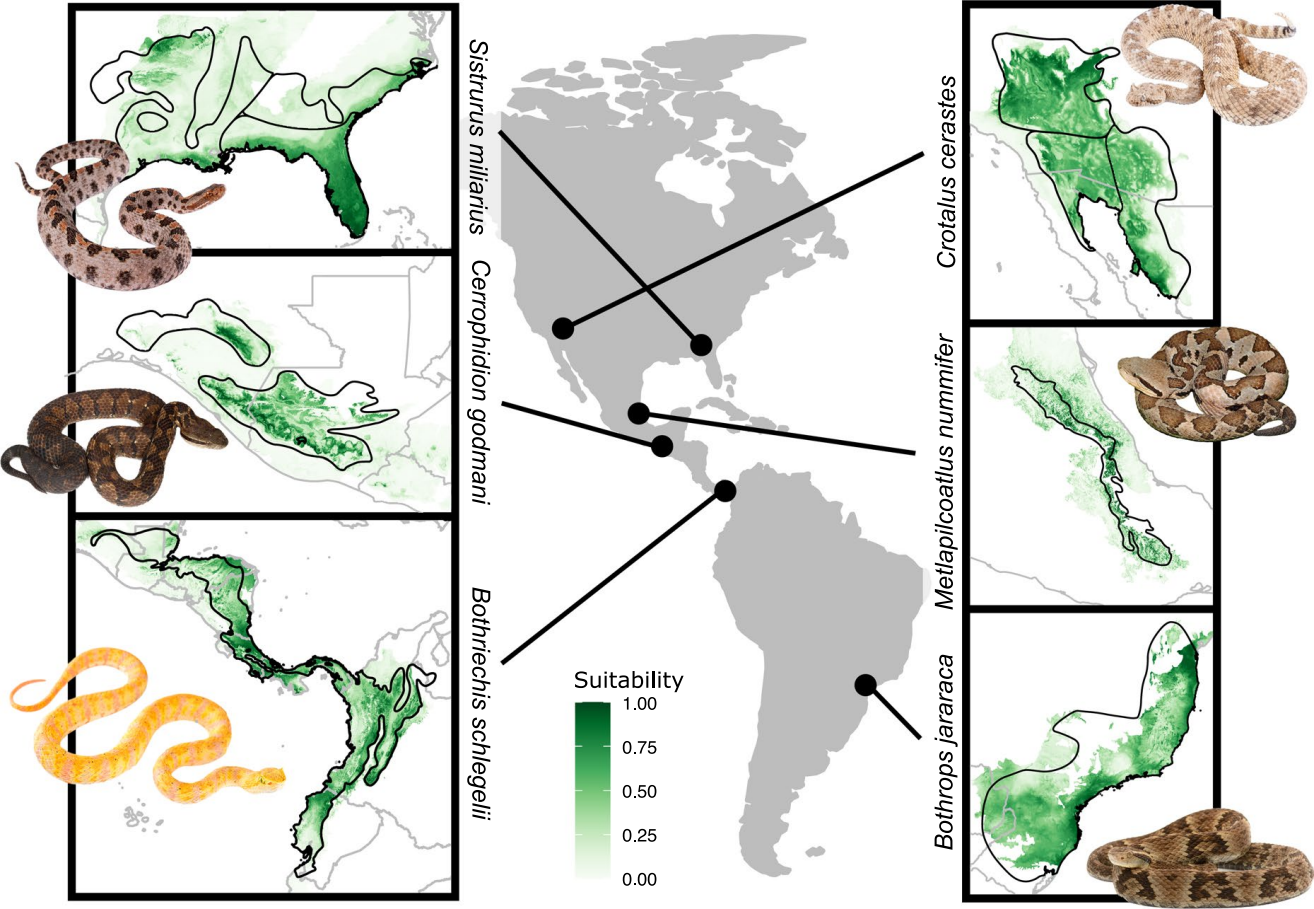
Distribution maps and SDMs of nine representative species. Distribution maps are outlined with black (subspecies boundaries displayed in *Crotalus cerastes* and *Sistrurus miliarius*). Photo Credit: *Sistrurus miliarius* and *Crotalus cerastes* (Tristan Schramer, Clemson University); *Cerrophidion godmani* and *Metlapilcoatlus nummifer* (Jason Jones, Herp.MX); *Bothriechis schlegelii* (Tropical Herping, tropicalherping.com); *Bothrops jararaca* (Welington Coelho).

Although topography variables often resulted in worse models, they likely facilitated micro-habitat refinement of the models. For example, including elevation variables into the final combination model for *Agkistrodon piscivorus* – an aquatic specialist – produced a model which closely followed riverine habitats and reduced presence in high-elevation habitats (Fig. 2). The combination models, therefore, more accurately traced the expected distribution and habitats for a given species. Finally, the SDMs demonstrated that the constructed distribution maps closely matched the optimal habitat for many species (Fig. 3; Fig. S2). Final SDM statistics are available in Supplementary Table 4.

## Usage Notes

Data, distributions, SDMs, and code can be accessed from the VenomMaps GitHub repository (github.com/RhettRautsaw/ VenomMaps) with stable releases archived on Zenodo^15^. To aid in public accessibility and utility, we developed a R Shiny app to view distribution maps, occurrence records, and SDMs (rhettrautsaw.app/shiny/VenomMaps). A user guide for the Shiny App is available on the GitHub repository. In the Shiny app, distributions can be filtered by country or available SDM. Additional information on maximum size of each species was compiled from Feldman *et al*.^75^ and is easily viewed in secondary tab for “General Information”.

## Supplementary information

Supplementary Table 1

Supplementary Table 2

Supplementary Table 3

Supplementary Table 4

Supplementary Figures

## Data Availability

All code, including custom scripts such as occ_cleaner, bind.tip2, SelectVariables, and autokuenm discussed above, are available as R scripts and summarized in Markdown format under the code directory in the GitHub project repository (github.com/RhettRautsaw/VenomMaps). We also provide functions to convert SDM outputs (R function: raw2clog, raw2log, log2raw) and threshold models (R function: raster_threshold) in the functions.R script.
