# Supplementary material for: VenomMaps: Updated species distribution maps and models for New World pitvipers (Viperidae: Crotalinae)
Source: Sci Data. 2022 May 25;9:232. doi: 10.1038/s41597-022-01323-4 (PMC9132920; doi:10.1038/s41597-022-01323-4)
Supplement: Supplementary file 5 — Supplementary Figures [file 41597_2022_1323_MOESM5_ESM.pdf]

Supplemental Figures

VenomMaps: Updated Species Distribution Maps and Models for New World Pitvipers (Viperidae: Crotalinae)

Rhett M. Rautsaw, Gustavo Jiménez-Velázquez, Erich P. Hofmann, Laura R. V. Alencar, Christoph I. Grünwald, Marcio Martins, Paola Carrasco, Tiffany M. Doan, Christopher L. Parkinson

List of Figures

Figure S1 2
Figure S2 3

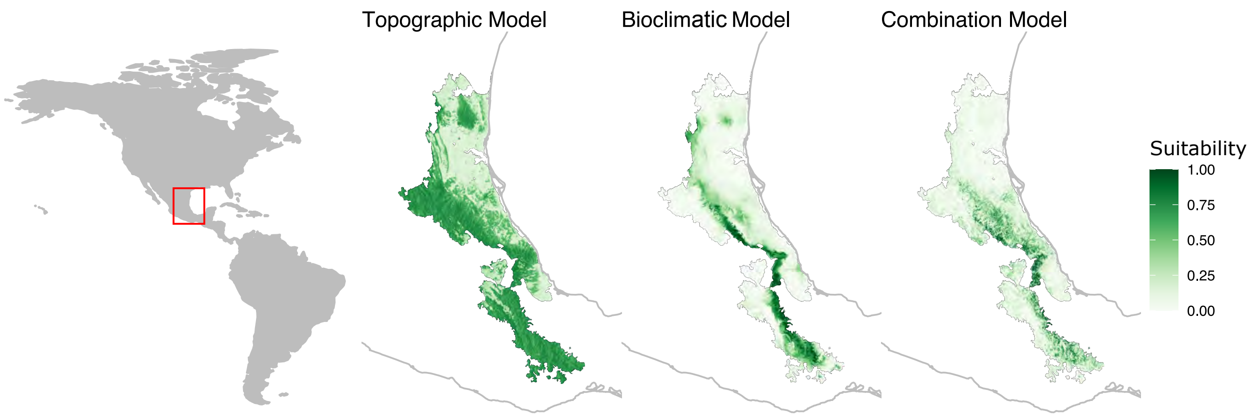

Figure S1. SDMs for *Metlapilcoatlus nummifer* demonstrate how topographic variables overestimate suitability across the entire modellling extent compared to bioclimatic variables or a combination of bioclimatic and topographic variables.

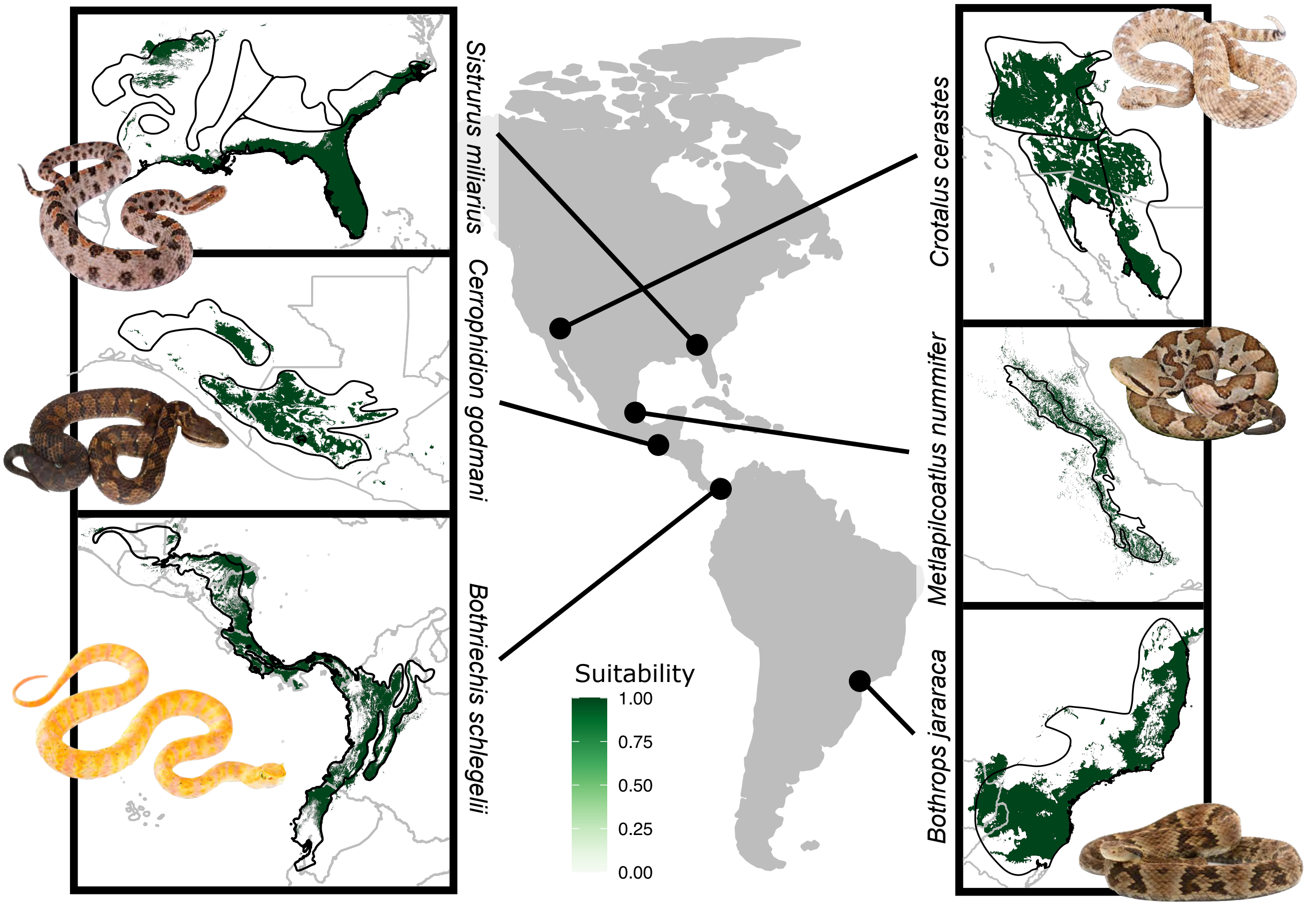

Figure S2. Distribution maps and 10th Percentile Training Threshold SDMs of nine representative species. Distribution maps are outlined with black (subspecies boundaries displayed in *Crotalus cerastes* and *Sistrurus miliarius*). Photo Credit: *Sistrurus miliarius* and *Crotalus cerastes* (Tristan Schramer, Clemson University); *Cerrophidion godmani* and *Metlapilcoatlus nummifer* (Jason Jones, Herp.MX); *Bothriechis schlegelii* (Tropical Herping, tropicalherping.com); *Bothrops jararaca* (Welington Coelho).
